# Sustained Release Formulation of Primaquine for Prevention of Relapse of *Plasmodium vivax* Malaria: A Randomized, Double-Blind, Comparative, Multicentric Study

**DOI:** 10.1155/2015/579864

**Published:** 2015-08-20

**Authors:** Anil Pareek, Nitin Chandurkar, Nithya Gogtay, Alaka Deshpande, Arjun Kakrani, Mala Kaneria, Partha Karmakar, Arvind Jain, Dhanpat Kochar, Arun Chogle, Arnab Ray

**Affiliations:** ^1^Medical Affairs and Clinical Research, Ipca Laboratories Limited, Mumbai 400067, India; ^2^Clinical Research & Development, Ipca Laboratories Limited, Mumbai 400067, India; ^3^Department of Clinical Pharmacology, Seth G. S. Medical College & K. E. M. Hospital, Mumbai 400012, India; ^4^Department of Medicine, Grant Medical College & Sir J. J. Group of Hospitals, Mumbai 400008, India; ^5^Department of Medicine, Padmashree Dr. D. Y. Patil Medical College, Pimpri, Pune 411018, India; ^6^Department of Medicine, B. Y. L. Nair Charitable Hospital & T. N. Medical College, Mumbai 400008, India; ^7^Department of Medicine, IPGMER and SSKM College & Hospital, Kolkata 700020, India; ^8^Department of Medicine, R. G. Kar Medical College & Hospital, 1 Kshudiram Bose Sarani, Kolkata 700004, India; ^9^Department of Medicine, Dr. S. N. Medical College, Jodhpur 342003, India; ^10^Kothari Medical and Research Institute, Bikaner 334004, India; ^11^Kasturba Hospital for Infectious Diseases, Mumbai 400011, India

## Abstract

*Background*. Primaquine is used to eradicate latent *Plasmodium vivax* parasite from liver, with administration of standard dose daily up to 14 days. We studied efficacy, safety, and tolerability of sustained release (SR) formulation of primaquine in comparison with conventional primaquine in preventing relapse of *P. vivax* malaria. *Methods*. Microscopically confirmed cases of *P. vivax* malaria received chloroquine therapy for three days. Aparasitemic and asymptomatic patients were then randomized to receive either conventional primaquine 15 mg for 14 days or primaquine SR 15 mg for 14 days, or primaquine SR 30 mg for seven days. *Results*. Of the 360 patients, who received chloroquine therapy, 358 patients were randomized. Two-hundred eighty-eight patients completed six-month follow-up and four patients (three: conventional primaquine 15 mg (2.86%), one: primaquine SR 30 mg (0.93%)) showed relapse confirmed by PCR genotyping. Drug compliance was significantly better in primaquine SR 30 mg group (95.57%, *p* = 0.039) without any serious adverse events. *Conclusion*. Primaquine SR 15 mg and primaquine SR 30 mg could be an effective alternative to conventional primaquine 15 mg due to their comparable cure rates and safety profile. Shorter treatment duration with primaquine SR 30 mg may increase patient compliance and may further reduce relapse rates. *Clinical Trial Registration*. This trial is registered with CTRI/2010/091/000245.

## 1. Introduction

*Plasmodium vivax* is the second most prevalent malaria species that is spread over a wider geographical area [1]. India contributes about 70% of malaria in the South East Asian Region of WHO [2].* P. vivax* is characterized by hypnozoite relapse in liver. These hypnozoites can become active months or even year later and can cause relapse after resolution of the primary illness [3].

As per WHO Guidelines, the current standard treatment for* P. vivax* is chloroquine monotherapy for three days followed by supplemental primaquine therapy as a single daily dose for 14 days for the purpose of eradicating dormant parasite in the liver and preventing relapses [4]. Primaquine is known to cause cardiac arrhythmia and prolongation of QT interval, mainly with high dose and hemolytic anemia especially in G6PD deficient individuals [5]. Several alternative compounds like tafenoquine, bulaquine, tinidazole, and imidazolidinone have been tested in various stages of clinical trials as a replacement for primaquine. However, data on safety with widespread use of bulaquine is not yet available. Also more studies are required to establish efficacy and safety of tafenoquine [6, 7]. Hence, primaquine remains a drug of choice for radical cure of malaria.

Primaquine has a narrow therapeutic range and a short elimination half-life of about four to seven hours. Thus, primaquine requires daily administration for up to 2 weeks that may result in poor compliance [8, 9]. This poor compliance may increase the chances of relapse after weeks, months, or years [6].

The concept of developing primaquine SR 15 mg (manufactured by Ipca Labortories Ltd., India) and primaquine SR 30 mg (manufactured by Ipca Labortories Ltd., India) formulations was based on maintaining adequate therapeutic concentration over 24 hours and reducing the treatment duration from 14 days to seven days for better patient compliance. The hypnozoitocidal activity of primaquine is reported to depend more on the total dose administered than on the length of the treatment [10]. Anvikar et al. have also mentioned that strategies to improve antirelapse primaquine treatment could include directly observed therapy or administering the same total dose over a short duration [11]. The hypothesis for this study was that, with primaquine SR 30 mg, compliance to the therapy will be improved because of reduction in treatment duration from 14 days to 7 days without compromising on its efficacy in preventing relapse of* P. vivax* malaria. Whereas, with primaquine SR 15 mg, therapeutic concentration will be maintained throughout 24 hours that may lead to reduction in relapse rate when compared with conventional primaquine therapy.

The objective of this study was to compare the efficacy, safety, and tolerability of primaquine SR 15 mg and primaquine SR 30 mg tablets with conventional primaquine 15 mg tablets in the prevention of relapse of* P. vivax* malaria and to check whether the duration of therapy can be reduced from 14 days to seven days with primaquine SR 30 mg tablets. Safety and tolerability of study medications were also assessed during the study.

## 2. Research Design and Methods

### 2.1. Participants

Patients of either sex, aged between 18 and 65 years, and body weight >40 kg were eligible for participation if they had microscopically confirmed* P. vivax* malaria with ≥1000 asexual parasites/*μ*L of blood, axillary temperature ≥37.5°C (≥99.5°F), and presence of at least five of the following signs and symptoms of uncomplicated malaria: chills, nausea, vomiting, headache, malaise, diarrhea, anorexia, abdominal cramps, myalgia, and arthralgia.

Patients with mixed malarial infections, severe or complicated malaria (as defined by WHO), G6PD deficiency, or any other significant concomitant illness were not included in the study. Patients with history of dark urine or significant hemoglobinuria related to previous primaquine treatment or those with history of methemoglobinemia were not included in the study. Patients with protracted vomiting and oliguria, or those with underlying condition compromising bone marrow function or having a tendency to granulocytopenia, were excluded from the study. Patients taking cardioactive drug or potentially hemolytic drugs or drugs that may interact with study drugs were not included in the study. Patients having history of hypersensitivity to any of the study related drugs and those on another investigational drug or history/presence of substance abuse were not included in the study. Pregnant or lactating women or women of child-bearing potential not using medically accepted means of birth control were also excluded from the study.

### 2.2. Study Design

This double-blind, double-dummy, randomized, comparative, and multicentric study was conducted at eight centers according to GCP Guidelines and the Declaration of Helsinki. All suspected malaria cases were provided with a patient information sheet in a language understood by the patient and/or patient's representative, as applicable. Only those patients who provided signed written informed consent were screened for the study. At screening, patients were evaluated for signs and symptoms of malaria. Blood and urine sample were collected to perform routine laboratory investigations. Patients satisfying study eligibility criteria were included in the study and were hospitalized for 3 days during which they received chloroquine therapy. Aparasitemic and asymptomatic patients after three days of chloroquine therapy were discharged from the hospital and were randomized in 1 : 1 : 1 ratio to receive conventional primaquine 15 mg (Group 1) or primaquine SR 15 mg (Group 2) or primaquine SR 30 mg (Group 3). Patients randomized to Group 1 received active conventional primaquine 15 mg for 14 days along with matching placebo of primaquine SR 15 mg and primaquine SR 30 mg. Patients randomized to Group 2 received active primaquine SR 15 mg for 14 days along with matching placebo of conventional primaquine 15 mg and primaquine SR 30 mg. Patients randomized to Group 3 received active primaquine SR 30 mg for 7 days and matching placebo of conventional primaquine 15 mg and primaquine SR 15 mg. For the remaining 7 days, patients from Group 3 received matching placebo of conventional primaquine 15 mg, primaquine SR 15 mg, and primaquine SR 30 mg. Randomization codes were generated using computer generated block randomization method. Patient specific sealed boxes of medicine were provided to each study site.

After discharge, patients were required to visit the hospital at Day 7, Day 14, Day 21, and Day 28 and then monthly for the next five months. Patients were instructed to visit the hospital immediately in case they became symptomatic at any other time during the study.

### 2.3. Outcome

The primary efficacy parameter was the absence of microscopically proven asexual forms of* P. vivax* after primaquine therapy. Follow-up time was measured from the first dose of primaquine to the date of relapse, withdrawal from the study, lost to follow-up, or completion of the study as per protocol. At screening visit, blood sample was collected on Whatman filter paper for PCR genotyping. Repeat sample was to be collected if the patient showed recurrence of parasitemia after initial clearance and completion of primaquine therapy. PCR genotyping was performed for such patients to allow differentiation between relapse and reinfection.

During chloroquine therapy, patients were hospitalized to allow evaluation of clinical signs and symptoms of malaria and to perform thick and thin blood smears examination every 12 hours. Axillary body temperature was measured at every six hrs. Each follow-up included a review of clinical signs and symptoms, peripheral blood smear examination, and body temperature recording.

Secondary efficacy parameter included compliance to study medication assessed based on patient diary and medication bottles returned by the patients. Patients who consumed the drug as prescribed were considered as compliant to the study medication.

Safety assessment was based on reported adverse events and changes in the laboratory parameters. Changes in the laboratory parameters were assessed by performing tests for routine hematology, biochemistry, and urinalysis at baseline and at the end of therapy. At screening visit, test to assess G6PD deficiency was performed for all patients and patients with G6PD deficiency were excluded from the study. Also, urine pregnancy test was performed for all female patients of child-bearing potential. Female patients with positive test result were excluded from the study. At each visit during the study, physical examination was performed and vital signs were recorded to perform safety assessments.

### 2.4. Statistical Methods

Sample size of 100 patients (without dropout) per treatment group was considered to give 80% power with 95% confidence interval (two-sided) (*α* = 0.05) for the maximum difference of 10% in relapse rates between treatment groups.

Efficacy was assessed using modified intention-to-treat (ITT) analysis that included all randomized patients who completed 14 days of primaquine therapy without any protocol violation. Last observation carried forward approach was used to impute missing assessments.

The demographic and initial clinical and biological characteristics of the patients were compared using descriptive statistics. Data was provided in the form of mean ± SD (range) for continuous variables and percentage for categorical variables. At baseline, all patients were compared using analysis of variance (ANOVA) for parametric variables or Kruskal-Wallis test for nonparametric continuous variables and chi-square or Fisher's exact test (if cell frequency is <5) for categorical variables for homogeneity.

The primary efficacy parameter was the absence of microscopically proven* P. vivax* malaria (asexual forms) after initial clearance and completion of primaquine therapy, which was calculated and compared using chi-square test or Fisher's exact test. Reduction in treatment duration from 14 days to seven days was the secondary efficacy parameter which was compared using *t*-test. Relapse rate (relapses per person-year) was calculated* post hoc* as the number of patients who had relapse divided by the total number of person-years of follow-up. Corresponding confidence interval (CI) based on the ratio of two Poisson variables was calculated using the exact conditional distribution. The Kaplan-Meier method was used to estimate the cumulative risk of relapse and was compared using Log rank test.

Safety population included all patients who gave written informed consent for participation in the study. Changes in laboratory parameters were evaluated by paired *t*-test, Wilcoxon signed rank test for within group, ANOVA or Kruskal-Wallis test as appropriate for between group analyses. The significance level was set as 0.05 for all statistical tests. All analyses were performed using SAS 9.2.

## 3. Results

### 3.1. Trial Subjects

**Figure 1:**
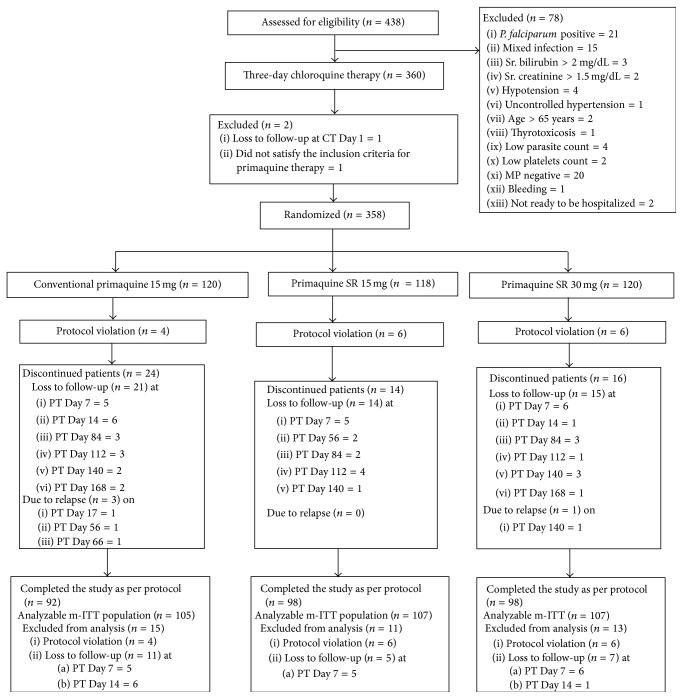
Disposition of study participants. PT: primaquine therapy, CT: chloroquine therapy, and MP: malarial parasite.

Of the 438 patients screened, 360 patients were included in the study to receive 3 days' chloroquine therapy. Of these 360 patients, one patient was lost to follow-up on Day 1 of chloroquine therapy and one patient did not satisfy the eligibility criteria for receiving primaquine therapy. The remaining 358 aparasitemic and asymptomatic patients were randomized to receive either conventional primaquine 15 mg for 14 days (120 patients) or primaquine SR 15 mg (118 patients) for 14 days or primaquine SR 30 mg (120 patients) for 7 days. The complete disposition of study participants is given in Figure 1.

Table 1 describes baseline demography and disease characteristics of patients.

There was a consistent reduction in mean parasite count throughout chloroquine therapy. At the end of chloroquine therapy, 99.4% of patients were aparasitemic and 97.8% of patients were afebrile (Table 2).

The mean PCT was 39.94 ± 16.54 hrs and mean FCT was 32.53 ± 18.96 hrs during the chloroquine therapy.

### 3.2. Efficacy Assessments

#### 3.2.1. Therapeutic Response during Follow-Up Visit

Of the 319 patients included in the m-ITT population for efficacy assessment, four patients (three patients from conventional primaquine 15 mg group (2.86%) and one from primaquine SR 30 mg group (0.93%)) returned to clinic with presence of parasitaemia and signs and symptoms of malaria. The PCR genotyping analysis confirmed the relapse of* P. vivax* malaria based on the presence of the same genotype that was present at baseline for respective patient. There was no statistically significant difference between three treatment groups in terms of % relapse rate. The apparent success rate (no subsequent appearance of* P. vivax* malaria) was >99% in primaquine SR 30 mg group. For all patients treated with primaquine SR 30 mg, the incidence (per person-year) of relapse was reduced by 68.2% (95% CI, −29.6 to 99.4), and with primaquine SR 15 mg, incidence of relapse was reduced by 100% (95% CI, −134 to 100) when compared to patients treated with conventional primaquine 15 mg. For patients receiving primaquine SR formulations (15 mg or 30 mg), the incidence of relapse was reduced by 83.9% (95% CI, −99.5 to 99.7) as compared to patients treated with conventional primaquine 15 mg (Table 3).

**Figure 2:**
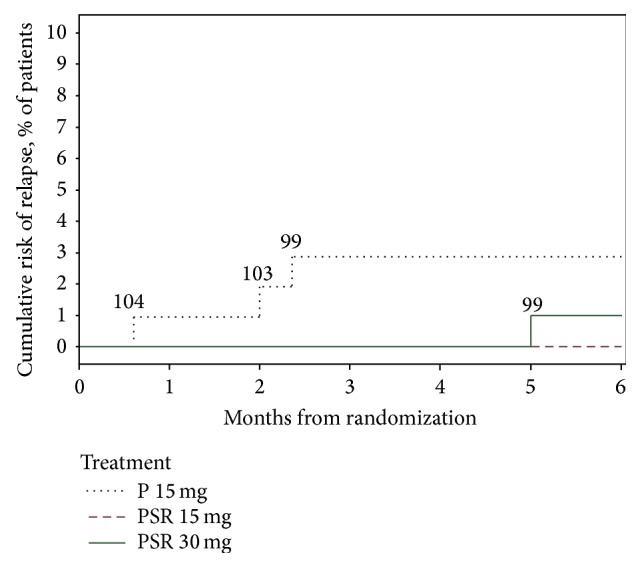
Kaplan-Meier estimates of cumulative risk of relapse. P 15 mg: conventional primaquine 15 mg, PSR 15 mg: primaquine sustained release 15 mg, and PSR 30 mg: primaquine sustained release 30 mg. Numbers written over the line are patients still undergoing follow-up. Differences in cumulative risk of relapse are statistically insignificant; *p* = 0.16 for test of equality over strata using Log rank test.

All four cases of relapse were considered treatment failure and were excluded from the study and treated as per investigator's discretion. The cumulative risk of relapse is summarized in Figure 2.

#### 3.2.2. Secondary Efficacy Parameter

Compliance to study medication was significantly better in primaquine SR 30 mg (95.57%) and primaquine SR 15 mg (95.5%) groups as compared to conventional primaquine 15 mg group (93.1%) (*p* value: conventional primaquine 15 mg versus primaquine SR 15 mg = 0.045, conventional primaquine 15 mg versus primaquine SR 30 mg = 0.039).

### 3.3. Safety Assessments

All the study drugs were well tolerated and there was no report of SAE. Commonly reported AEs during primaquine therapy and subsequent follow-up were headache, malaise, nausea, fever, and myalgia (Table 4). Twenty-one patients (five patients from primaquine 15 mg, 10 patients from primaquine SR 15 mg, and six patients from primaquine SR 30 mg group) reported 39 AEs during primaquine therapy and follow-up visits (Table 4). None of the patients from primaquine SR 30 mg group showed intolerance or dose-related side effects.

## 4. Discussion and Overall Conclusion

In the present study, sustained release formulation of primaquine was studied for the first time for preventing relapse of* P. vivax* malaria. From the time of its discovery, primaquine is the only available drug for radical cure of* P. vivax* malaria; however, short half-life, rapid metabolism, narrow therapeutic range, and requirement of daily administration for up to 14 days limit the use of primaquine. The long duration of treatment of primaquine sometimes results in poor compliance by the patients and may lead to relapse of malaria days, months, or years later. Sustained release formulations of primaquine were prepared to maintain adequate drug concentration throughout treatment period and to reduce treatment duration from 14 days to 7 days. This short duration and sustained release of drug from the formulation could have less toxic effects with maximal eradication of liver stages of* P. vivax* parasite.

Experimental studies with different strains of* Plasmodium* have shown that the dose of 15 mg/day is ineffective in preventing relapses. Primaquine 15 mg/day was found ineffective in preventing relapses even with good compliance [6].

A clinical study conducted in Thailand [7] and a report published by Bunnag et al. showed improvement in cure rate when primaquine was used at a daily dose of 22.5 mg for 14 days [12]. In an article published by Baird and Rieckmann, a dose regimen of 30 mg daily for 14 days (420 mg total dose) was widely recommended in Southeast Asia and Southwest Pacific region due to good efficacy [13]. In a trial of oral artesunate with or without high dose of primaquine for the treatment of* P. vivax* malaria in Thailand, primaquine with higher dose (0.6 mg base/kg/day) for 14 days was highly effective [14]. In all the cases, immediate release formulation of primaquine was used and patient had to consume primaquine compulsorily for 14 days. Attempts have been made wherein high dose of 0.6 mg base/kg/day of primaquine when administered in various regimens ranging in duration from five days to 14 days after artesunate therapy was well tolerated and equivalent or superior to the standard regimen of primaquine therapy [15]. Preclinical studies in nonhuman primates showed that the total dose of primaquine, rather than the schedule of treatment, determined the efficacy of treatment [16]. Thus, we developed a formulation of primaquine 30 mg that required administration for 7 days (total dose 210 mg).

The results of present study indicate that both primaquine SR 15 mg and primaquine SR 30 mg formulations are equally effective as that of primaquine 15 mg in terms of preventing relapse of* P. vivax* malaria. Further, there was increased patient compliance to the sustained release formulation and the drug compliance was significantly better in primaquine SR 30 mg and primaquine SR 15 mg groups as compared to conventional primaquine 15 mg group (conventional primaquine 15 mg versus primaquine SR 15 mg, *p* = 0.045; conventional primaquine 15 mg versus primaquine SR 30 mg, *p* = 0.039). The reason for significantly better compliance with primaquine SR 30 mg is reduction in treatment duration from 14 days to 7 days. In our study, total 23 patients were lost to follow-up up to Day 14. Of these 23 patients, 16 patients were lost to follow-up between Day 1 and Day 7 of primaquine treatment, whereas 7 patients were lost to follow-up between Day 8 and Day 14. Thus, we may assume that if the duration of primaquine therapy is reduced to 7 days, 30.43% (7 of 23 patients who were lost to follow-up between Day 8 and Day 14) of total lost to follow-up cases would have completed the primaquine therapy as required. Thus, by administering the total effective dose in the shortest possible duration, compliance to primaquine therapy may be possibly increased without compromising on its efficacy in preventing relapse of* P. vivax* malaria. The AEs reported in this study were the same as that generally expected in malaria patients with antimalarial medications. There were no dose-related toxic effects even in primaquine SR 30 mg group when compared with those observed in conventional primaquine therapy groups. All the study drugs were well tolerated and all the patients showed overall good response to the therapies.

In conclusion, the results of the present study demonstrated comparable efficacy, safety, and tolerability of primaquine SR 15 mg and primaquine SR 30 mg with that of conventional regimen of primaquine 15 mg in the prevention of relapse of* P. vivax* malaria. Administration of primaquine SR 30 mg will not only reduce the treatment duration but also increase the patient compliance that will help in radical cure of* vivax* parasite. Primaquine SR 15 mg formulation has also promising prospect when compared to conventional primaquine therapy with reference to number of relapse cases, may be because of sustained therapeutic concentration that was maintained throughout 24 hours. This indicates that SR formulation of primaquine could be a novel, effective, and safe alternative to long-established conventional primaquine therapy in the prevention of relapse of* P. vivax* malaria.

## Figures and Tables

**Table 1:** Demographic and baseline disease characteristics of patients.

Parameters	P 15 mg (*N* = 120)	PSR 15 mg (*N* = 118)	PSR 30 mg (*N* = 120)	Total^a^ (*N* = 358)	*p* value
Male^b^	99 (82.5)	99 (83.9)	98 (81.7)	296 (82.7)	0.923

Age (years)^c^	31 (23)	28 (21)	28 (18)	29 (20)	0.197

Weight (kg)^d^ Min–max	56.77 ± 9.07	56.41 ± 9.36	57.13 ± 8.84	56.77 ± 9.07	0.760
	40–86	32–92	40–86	32–92	

Body temperature^d^ (°C)	38.50 ± 0.84	38.5 ± 0.93	38.49 ± 0.76	38.5 ± 0.84	0.988

Respiration rate^d^ (breaths/min)	17.85 ± 3.74	17.98 ± 3.78	17.98 ± 3.79	17.95 ± 3.76	0.943

Parasite density^d^ (/*µ*L) (Min–Max)	6564.9 ± 12614.9	6168.7 ± 15128.1	6145.9 ± 13508.3	6293.9 ± 13744.5	0.969
	1000–100000	1000–141300	800–121100	800–141300	

Patients with parasites^b^					
<5000/*µ*L	90 (75.0)	92 (78.0)	95 (79.2)	277 (77.4)	0.986
≥5000 and <15000/*µ*L	16 (13.3)	15 (12.7)	14 (11.7)	45 (12.6)	
≥15000 and <30000/*µ*L	10 (8.3)	8 (6.8)	7 (5.8)	25 (6.9)	
≥30000/*µ*L	4 (3.3)	3 (2.5)	4 (3.3)	11 (3.1)	

Signs and symptoms^b^					
Fever	120 (100.0)	118 (100.0)	119 (99.17)	357 (99.7)	0.664
Chills	118 (98.33)	117 (99.15)	118 (98.33)	353 (98.5)	0.999
Headache	116 (96.67)	113 (95.76)	110 (91.67)	338 (94.6)	0.207
Nausea	99 (82.5)	88 (74.58)	89 (74.17)	276 (77.0)	0.209
Malaise	89 (74.17)	81 (68.64)	89 (74.17)	258 (72.2)	0.576
Vomiting	59 (49.17)	57 (48.31)	58 (48.33)	174 (48.4)	0.981
Myalgia	57 (47.5)	59 (50.0)	57 (47.5)	173 (48.3)	0.929
Anorexia	59 (49.17)	52 (44.07)	57 (47.5)	167 (46.7)	0.729
Arthralgia	11 (9.2)	17 (14.4)	14 (11.8)	42 (11.7)	0.461
Abdominal cramps	9 (7.5)	9 (7.6)	8 (6.7)	26 (7.3)	0.999
Diarrhoea	6 (5.0)	6 (5.1)	5 (4.2)	17 (4.7)	0.953

P 15 mg: conventional primaquine 15 mg, PSR 15 mg: primaquine sustained release 15 mg, and PSR 30 mg: primaquine sustained release 30 mg.

^a^Two patients were excluded from the total number of patients enrolled to receive chloroquine therapy.

b indicates value shown as *n* (%), Chi-square test used for comparison.

c indicates value shown as median (IQR), Kruskal-Wallis test used for the comparison.

d indicates value shown as mean ± SD, one way ANOVA used for comparison.

**Table 2:** Parasite clearance and fever clearance in number of patients during chloroquine therapy.

Time point	Parasite clearance *n* (%)	Fever clearance *n* (%)
At 6 hrs	NA	122 (33.9)
At 12 hrs	35 (9.7)	150 (41.7)
At 18 hrs	NA	159 (44.2)
At 24 hrs	117 (32.5)	173 (48.1)
At 30 hrs	NA	163 (45.3)
At 36 hrs	222 (61.7)	241 (66.9)
At 42 hrs	NA	284 (78.9)
At 48 hrs	281 (78.1)	314 (87.2)
At 54 hrs	NA	325 (90.3)
At 60 hrs	325 (90.3)	345 (95.8)
At 66 hrs	NA	349 (96.9)
At 72 hrs	358 (99.4)	352 (97.8)
At 78 hrs	NA	357 (99.2)
At 84 hrs	NA	358 (99.4)

The data includes all patients who were included to receive chloroquine therapy, NA: not applicable values.

**Table 3:** Efficacy of PSR 15 mg and PSR 30 mg against relapse of *P. vivax* malaria.

Characteristics	P 15 mg	PSR 15 mg	PSR 30 mg	PSR 15 mg + PSR 30 mg
Number of randomized patients	120	118	120	238

Duration of follow-up (days)				
Mean	142.3	149.6	149.0	149.3
Median	168	168	168	168
Total	17076	17652	17876	35528

Number of relapse, *n* (%)^a^	3 (2.86)	0 (0.0)	1 (0.93)	1 (0.42)

*p* value^b^	—	0.076	0.293	0.069

Relapses per persons-year^c^	0.064	0.00	0.02	0.01

Reduction in incidence, % (95% CI)^d^	NA	100 (−134 to 100)	68.2 (−29.6 to 99.4)	83.9 (−99.5 to 99.7)

*p* value^e^		0.059	0.176	0.057

P 15 mg: conventional primaquine 15 mg, PSR 15 mg: primaquine sustained release 15 mg, and PSR 30 mg: primaquine sustained release 30 mg.

^a^Denominator used is the number of patients who had completed treatment period of 14 days (for P 15 mg, *N* = 105; for PSR 15 mg, *N* = 107; and for PSR 30 mg, *N* = 107).

^b^PSR 15 mg and PSR 30 mg compared with P 15 mg conventional using Fisher's exact test.

^c^Denominator used is the total duration of follow-up in years (calculated based on total number of follow-up days).

^d^Value shown is a conditional exact 95% CI for ratio of two Poisson variables.

^e^Percentage reduction in incidence rate of PSR 15 mg and PSR 30 mg compared with conventional P 15 mg.

NA: not applicable value.

**Table 4:** Numbers of AEs reported during chloroquine therapy and primaquine therapy.

Adverse event	Chloroquine therapy total (*N* = 360)	Primaquine therapy			
		P 15 mg (*N* = 120)	PSR 15 mg (*N* = 118)	PSR 30 mg (*N* = 120)	Total (*N* = 358)
Malaise	2	3	4	1	8
Headache	4	1	2	2	5
Nausea	12	2	1	1	4
Decreased appetite	8	0	3	0	3
Pyrexia	1	1	1	1	3
Vomiting	5	1	1	1	3
Dizziness	0	0	1	1	2
Myalgia	0	2	0	0	2
Asthenia	0	0	1	1	2
Arthralgia	0	0	1	0	1
Pruritus	5	0	0	1	1
Haemoglobin decreased	0	0	0	1	1
Muscle spasms	0	0	1	0	1
Dyspnoea exertional	0	0	1	0	1
Dyspepsia	0	0	0	1	1
Diarrhoea	0	0	1	0	1
Diarrhoea	1	0	0	0	0
Cough	1	0	0	0	0
Total events	**39**	**10**	**18**	**11**	**39**
Total patients, *n* (%)	**23 (6.4)**	**5 (4.2)**	**10 (8.5)**	**6 (5.0)**	**21 (5.9)**

P 15 mg: conventional primaquine 15 mg, PSR 15 mg: primaquine sustained release 15 mg, and PSR 30 mg: primaquine sustained release 30 mg.

*p* value: P 15 mg versus PSR 15 mg (*p* = 0.17), P 15 mg versus PSR 30 mg (*p* = 0.28), and PSR 15 mg versus PSR 30 mg (*p* = 0.77) (for patients reporting adverse events).
